# Multi-omics approaches for deciphering the complexity of traditional Chinese medicine syndromes in stroke: A systematic review

**DOI:** 10.3389/fphar.2022.980650

**Published:** 2022-09-06

**Authors:** Tingting Liu, Mingzhen Qin, Xuejiao Xiong, Xinxing Lai, Ying Gao

**Affiliations:** ^1^ Institute for Brain Disorders, Beijing University of Chinese Medicine, Beijing, China; ^2^ Department of Neurology, Dongzhimen Hospital, Beijing University of Chinese Medicine, Beijing, China; ^3^ Beijing University of Chinese Medicine, Beijing, China; ^4^ Chinese Medicine Key Research Room of Brain Disorders Syndrome and Treatment of the National Administration of Traditional Chinese Medicine, Beijing, China

**Keywords:** stroke, traditional Chinese medicine, syndromes, multi-omics, systematic review

## Abstract

**Background:** Deciphering the biological basis of traditional Chinese medicine (TCM) syndromes in complex diseases is challenging. Rapid advances in multi-omics approaches provide new opportunities to unveil the biological basis of TCM syndromes. We intend to summarize the latest significant progress and highlight the crucial value of applying multi-omics approaches to reveal TCM syndromes of stroke in a new horizon.

**Methods:** We systematically searched PubMed, EMBASE, Web of Science Core Collection (WOSCC), Cochrane Library, China National Knowledge Infrastructure (CNKI), Chinese Science and Technology Periodical Database (VIP), Wanfang database and China Biology Medicine Database (SinoMed) for relevant studies from their inception to 31 March 2022, and conducted a comprehensive systematic review (PROSPERO registration number: CRD42021285922).

**Results:** A total of 43 relevant studies were included in the final systematic review, genomics, transcriptomics, proteomics, and metabolomics were all involved. Some gene polymorphisms, differential lncRNAs, mRNAs, miRNAs, proteins, and metabolites may be associated with TCM syndromes of stroke. In addition, some studies conducted a preliminary exploration on the different diseases with the same TCM syndrome. The results showed that thioredoxin-dependent peroxidase reductase may be the specific marker protein of Liver-yang transforming into wind syndrome, and the network formed by mir-146b-5p, -199a-5p, and 23 targeted mRNAs may be the biomarker of Blood-stasis syndrome.

**Conclusion:** Multi-omics technologies have served as powerful tools to investigate the complexity of TCM syndromes and may hold the promise of promoting the modernization of TCM as well as personalized medicine of TCM in stroke.

## 1 Introduction

Traditional Chinese medicine (TCM) has accumulated valuable medical experience over thousands of years, which has played an important role in maintaining people’s health (Cheung, 2011). TCM syndrome, as one of the key concepts in TCM, is a generalization of the cause, location, nature, and developmental tendency of a disease at a specific stage and is identified through a comprehensive analysis of the clinical symptoms and signs gathered by a practitioner using inspection, auscultation, olfaction, interrogation, and palpation of the pulses (Wang and Xu, 2014; Wang and Dong, 2017). Usually, the same disease has variable TCM syndromes. Correctly identifying the TCM syndrome is the cornerstone for TCM practitioners to understand diseases and guide individualized clinical medication (Wang and Xu, 2014). However, TCM syndrome differentiation has a certain degree of complexity, ambiguity, and subjectivity (Jiang et al., 2012; Gu and Chen, 2014). Deciphering the biological basis of TCM syndromes will be conducive to objectively diagnosing syndromes, discovering the potential targets of Chinese herbal medicine, and ultimately leading to the discovery of new therapeutic drugs and promoting precision medicine in TCM.

In recent years, a series of studies have been conducted to elucidate the biological basis of TCM syndromes using low-throughput sequencing methods; however, this is insufficient due to the complexity of TCM syndromes (Hou et al., 2014; Liu et al., 2014; Han et al., 2015). With the advent of systems biology era, high-throughput, high-content genomics, transcriptomics, proteomics, and metabolomics methods, combined with robust bioinformatics and computational tools have been widely and effectively applied in the biological basis research of TCM syndromes, which have provided unparalleled information about quantities and interactions of different biomolecules at the system and whole organism levels (Gu and Chen, 2014; Guo et al., 2020). Simultaneously, considerable achievements have been made, such as the biological basis of two TCM syndromes (Cold-congealing and qi-stagnation, Qi-stagnation and blood-stasis) in coronary heart disease, transcriptomic research on two TCM syndromes (Spleen-qi deficiency, Spleen-stomach damp-heat) in chronic atrophic gastritis, and the dynamic biological network of TCM syndromes in chronic hepatitis B (Lu et al., 2019; Wu et al., 2021; You et al., 2021).

It is worth noting that stroke, as the second leading cause of death in the world and ranks first in China, has made great advances in understanding the pathophysiology with omics technology, generating a large amount of data and information at the multi-omics level (Collaborators, 2021). It is encouraging that potential biomarkers associated with etiological classification and prognosis, as well as new therapeutic targets, have been identified (Montaner et al., 2020). Notably, the application of multi-omics technology has also provided new opportunities for the elucidation of TCM syndromes for stroke, including metabolomics research on Phlegm-heat syndrome and Blood-stasis syndrome, as well as transcriptomics research on Yin syndrome and Yang syndrome (Cha et al., 2013; Cha et al., 2015; Zhao et al., 2019).

Considering that there is no comprehensive review on the current status of applying multi-omics approaches to reveal the biological basis of TCM syndromes in stroke, we conducted this systematic review to summarize the related progress, analyze the challenges that need to be addressed, and provide important insights into the biological complexity of TCM syndromes for future research.

## 2 Methods

This systematic review was registered in PROSPERO (CRD42021285922).

### 2.1 Search strategy and selection criteria

Eight electronic databases were searched without language limitation (from their inception to 31 March 2022): PubMed, EMBASE, Web of Science Core Collection (WOSCC), Cochrane Library, China National Knowledge Infrastructure (CNKI), Chinese Science and Technology Periodical Database (VIP), Wanfang database, and China Biology Medicine Database (SinoMed). All searches were conducted by combining free-text and MESH terms, including *stroke, omics, genomics, transcriptomics, proteomics, metabolomics, multi-omics, syndrome, ZHENG,* and *traditional Chinese medicine* (Supplementary Table S1)*.*

Two reviewers (MQ and XX) independently screened titles and abstracts and selected potential full texts for further analysis. Studies that fulfilled our pre-defined eligibility criteria were included in the review. Any disagreements were resolved through consensus or consultation with a third reviewer (XL). The detailed inclusion criteria were as follows: 1) stroke patients with specific TCM syndromes, 2) application of omics approaches to study TCM syndromes of stroke, and 3) experimental or observational studies. The exclusion criteria were as follows: 1) abstracts, editorials, letters, comments, case reports, and review papers; 2) articles on subarachnoid hemorrhage; and 3) intracerebral hemorrhage (ICH) caused by traumatic injury.

### 2.2 Data extraction

Data were independently extracted by two reviewers (MQ and XX) using a preformulated data collection form. A narrative summary of the results was produced according to specific data subjects: 1) the article’s author and publication year; 2) study characteristics, including the study site, disease, TCM syndromes, sample size, stroke onset time, omics type, omics technology, and specimen; and 3) the main findings. For each study, all relevant data were extracted from the tables, figures, text, and supplemental materials.

### 2.3 Quality assessment

The quality of included studies was assessed using an 11-item checklist recommended by the Agency for Healthcare Research and Quality. If an item was answered with “No” or “Unclear,” the item was scored as “0;” if the answer was “Yes,” the item was scored as “1.” Based on the total score (ranging from 0 to 11 points), the quality of the study was divided into high (8–11 points), fair (4–7 points), or low (< 4 points) (Hu et al., 2015; Zeng et al., 2015).

## 3 Results

**FIGURE 1:**
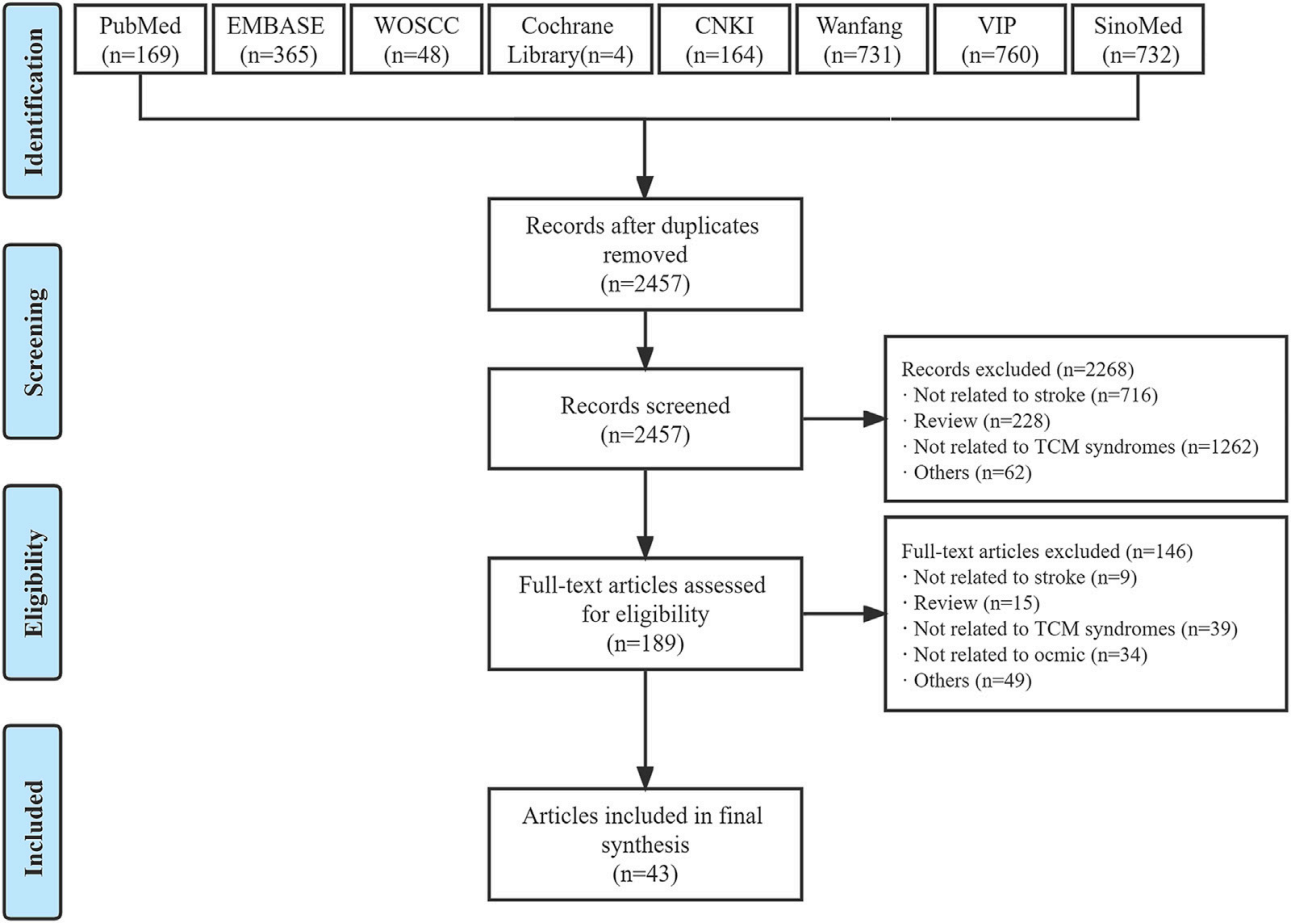
Flow diagram of study selection. CNKI, China National Knowledge Infrastructure; TCM, traditional Chinese medicine; VIP, Chinese Science and Technology Periodical Database; WOSCC, Web of Science Core Collection.

Our systematic search yielded 2973 studies through eight electronic databases, 516 of which were excluded after duplication and 2268 were excluded after abstract review. A total of 43 relevant studies were included in the final data set after a full-text review (Figure 1) (Xiong et al., 2007; Huang et al., 2008; Jia et al., 2008; Xiao et al., 2008; Zeng et al., 2008; Zhao et al., 2008; Hu et al., 2009; Xiong et al., 2011; Shang et al., 2012; Wang et al., 2012; Cha et al., 2013; Chen et al., 2013; Xie et al., 2013; Li et al., 2014; Yang et al., 2014; Cha et al., 2015; Shen et al., 2015; Gu et al., 2016a; Gu et al., 2016b; Gu et al., 2016c; Gu et al., 2016d; Huo and Tan, 2016; Liao et al., 2016; Wang et al., 2016; Gu et al., 2017; Zhao et al., 2018; Gu et al., 2019a; Gu et al., 2019b; Gu et al., 2019c; Gu et al., 2019d; Li et al., 2019; Liu et al., 2019; Wei et al., 2019; Yang et al., 2019; Zhang et al., 2019; Zhao et al., 2019; Zhu et al., 2019; Gu et al., 2020a; Gu et al., 2020b; Rong and Li, 2020; Zhang et al., 2020; Gu et al., 2021; Li et al., 2022).

Overall, 43 studies ranged in size from eight to 1802 participants, of which 41 studies were conducted in China (Xiong et al., 2007; Huang et al., 2008; Jia et al., 2008; Xiao et al., 2008; Zeng et al., 2008; Zhao et al., 2008; Hu et al., 2009; Xiong et al., 2011; Shang et al., 2012; Wang et al., 2012; Chen et al., 2013; Xie et al., 2013; Li et al., 2014; Yang et al., 2014; Shen et al., 2015; Gu et al., 2016a; Gu et al., 2016b; Gu et al., 2016c; Gu et al., 2016d; Huo and Tan, 2016; Liao et al., 2016; Wang et al., 2016; Gu et al., 2017; Zhao et al., 2018; Gu et al., 2019a; Gu et al., 2019b; Gu et al., 2019c; Gu et al., 2019d; Li et al., 2019; Liu et al., 2019; Wei et al., 2019; Yang et al., 2019; Zhang et al., 2019; Zhao et al., 2019; Zhu et al., 2019; Gu et al., 2020a; Gu et al., 2020b; Rong and Li, 2020; Zhang et al., 2020; Gu et al., 2021; Li et al., 2022), and two in South Korea (Cha et al., 2013; Cha et al., 2015). Of these, 32 studies enrolled patients with ischemic stroke (IS) (Huang et al., 2008; Jia et al., 2008; Zeng et al., 2008; Hu et al., 2009; Shang et al., 2012; Wang et al., 2012; Cha et al., 2013; Xie et al., 2013; Li et al., 2014; Cha et al., 2015; Shen et al., 2015; Gu et al., 2016a; Gu et al., 2016b; Gu et al., 2016c; Gu et al., 2016d; Huo and Tan, 2016; Liao et al., 2016; Wang et al., 2016; Gu et al., 2017; Gu et al., 2019a; Gu et al., 2019b; Gu et al., 2019c; Gu et al., 2019d; Liu et al., 2019; Zhao et al., 2019; Zhu et al., 2019; Gu et al., 2020a; Gu et al., 2020b; Rong and Li, 2020; Zhang et al., 2020; Gu et al., 2021; Li et al., 2022), nine with ICH (Xiong et al., 2007; Xiao et al., 2008; Zhao et al., 2008; Chen et al., 2013; Yang et al., 2014; Zhao et al., 2018; Li et al., 2019; Yang et al., 2019; Zhang et al., 2019), one with stroke complications (Wei et al., 2019), and one with both IS and ICH (Tables 1–T4) (Xiong et al., 2011). Genomics, transcriptomics, proteomics, and metabolomics were involved in the omics approaches (Table 5). The quality of all the studies was fair (Supplementary Table S2)*.*

**TABLE 1:** Summary of the main findings of the included genomics studies.

Source	Sites	Disease	TCM syndromes	Sample size	Onset time	Specimen	Technology	Main findings
Genomics								
Jia et al. (2008)	China	IS	Phlegm; Qi-deficiency	226	≤7 days	Plasma	ARMS-PCR	Angiotensin converting enzyme gene D allele may be related to Phlegm, I allele may be related to Qi-deficiency
Huang et al. (2008)	China	IS	Hyperactive liver-yang; Wind-phlegm and blood-stasis	104	NA	PVB	PCR-RFLP	Angiotensinogen gene M235T No correlation with TCM syndromes
Hu et al. (2009)	China	IS	Blood-stasis	227	NA	PVB	PCR-RFLP	Methylenetetrahydrofolate reductase gene C677T TT genotype may be related to Blood-stasis
Shang et al. (2012)	China	IS	Phlegm-heat and fu-organ excess; Qi-deficiency and blood-stasis; Wind-phlegm and fire-hyperactivity; Wind-phlegm stagnation; Wind-stirring due to yin-deficiency	390	NA	PVB	PCR-RFLP	Platelet membrane glycoprotein Iba·VNTRBC CD genotype may be related to Qi-deficiency and blood-stasis; BC, BD, AC genotype and B allele may be related to Wind-phlegm stagnation·HPA-2 No correlation with TCM syndromes
Xie et al. (2013)	China	IS	Blood-stasis; Phlegm-stasis	340	NA	PVB	TaqMan	Chromosome 12p13 rs12425791 No correlation with TCM syndromes
Shen et al. (2015)	China	IS	Qi-deficiency and blood-stasis	889	NA	Leucocyte	Sequenom MassARRAY	rs2107595 May be related to blood lipid metabolism of patients with Qi-deficiency and blood-stasis
Huo and Tan, (2016)	China	IS	Hyperactive liver-yang; Qi-deficiency and blood-stasis; Wind-stirring due to yin-deficiency; Wind-phlegm obstructing the meridians	110	<7 days	PVB	PCR-RELP	Fibrinogen β-148C/T gene May be related to the fibrinogen level of TT genotype carriers with Wind-phlegm obstructing the meridians
Wang et al. (2016)	China	IS	Qi-deficiency and blood-stasis; Wind-phlegm stagnation	1200	NA	PVB	Sequenom MassARRAY	Toll-like receptor 5 gene rs5744174
								The recessive model may be related to Wind-phlegm stagnation
Gu et al. (2016a)	China	IS	Wind-phlegm stagnation	916	NA	PVB	Sequenom MassARRAY	Histone deacetylase 9 gene rs2107595 May be related to blood lipid metabolism of patients with Wind-phlegm stasis
Gu et al. (2016b)	China	IS	Qi-deficiency and blood-stasis; Wind-phlegm stagnation	1200	≤7 days	PVB	Sequenom MassARRAY	MAP2K1 gene
								·rs9340
								May be related to Wind-phlegm stagnation in men, and levels of TG in patients with Qi-deficiency and blood-stasis
								·rs6928
								May be related to levels of TG and HDL in patients with Qi-deficiency and blood-stasis
								MAPK4 gene
								No correlation with TCM syndromes
Gu et al. (2016c)	China	IS	Qi-deficiency and blood-stasis; Wind-phlegm stagnation	1200	≤7 days	PVB	Sequenom MassARRAY	Toll-like receptor 7 gene rs2897827
								May be related to Wind-phlegm stagnation
Gu et al. (2016d)	China	IS	Qi-deficiency and blood-stasis; Wind-phlegm stagnation	1200	NA	PVB	Sequenom MassARRAY	Myeloid differentiation factor 88 gene rs7744
								May be related to blood lipid metabolism in patients with Wind-phlegm stagnation
Gu et al. (2017)	China	IS	Qi-deficiency and blood-stasis; Wind-phlegm stagnation	1200	NA	PBMCs	Sequenom MassARRAY	Tumor necrosis factor receptor-associated factor 6 gene
								·rs5030416
								The recessive model may be related to Wind-phlegm stagnation
								·rs5030411
								May engage in the inflammatory reaction of patients with Wind-phlegm stagnation
Zhu et al. (2019)	China	IS	Blood-stasis; Phlegm; Wind	527	NA	PVB	Sanger	Histone deacetylase 9 gene rs2240419
								No correlation with TCM syndromes
Gu et al. (2019a)	China	IS	Phlegm-stasis	1100	NA	PVB	Sequenom MassARRAY	Signal transducer and activator of transcription 5 gene rs319502
								May be related to coagulation and inflammatory reaction in patients with Phlegm-stasis
Gu et al. (2020a)	China	IS	Blood-stasis; Fire-heat; Phlegm; Qi-deficiency; Wind	1802	≤7 days	PVB	Sequenom MassARRAY	Selenocysteine insertion sequence binding protein 2 gene rs3211703
								May be related to 2 TCM syndromes (Blood-stasis, Qi-deficiency)
Zhang et al. (2020)	China	IS	Phlegm-heat and fu-organ excess; Qi-deficiency and blood-stasis; Wind-stirring due to yin-deficiency; Wind-phlegm obstructing the meridians	70	NA	PVB	NA	CYP2C19 gene
								CYP2C19 * 2 gene mutation may be related to Qi-deficiency and blood-stasis
Gu et al. (2021)	China	IS	Blood-stasis; Fire-heat; Phlegm; Qi-deficiency; Wind	1756	≤72 h	PVB	Sequenom MassARRAY	DNA polymerase kappa gene rs5744724
								May be related to 2 TCM syndromes (Blood-stasis, Qi-deficiency)
Gu et al. (2019b)	China	IS; CHD	Phlegm-stasis	1650	NA	PVB	Sequenom MassARRAY	Coagulation factor X gene
								·rs3093261
								May be related to coagulation function in Phlegm-stasis both IS and CHD
								·rs563964
								No correlation with TCM syndromes
Gu et al. (2019c)	China	IS; CHD	Phlegm-stasis	1650	NA	PVB	Sequenom MassARRAY	EP300gene rs20551
								May be related to coagulation function in Phlegm-stasis both IS and CHD
Gu et al. (2019d)	China	IS; CHD	Phlegm-stasis	1650	NA	PVB	Sequenom MassARRAY	Kinase insert domain receptor gene rs2305948, rs2239702
								May be related to the coagulation function in Phlegm-stasis both IS and CHD
Gu et al. (2020b)	China	IS, CHD	Phlegm-stasis	1650	NA	PVB	Sequenom MassARRAY	Decorin gene rs7441
								May be related to the lipid metabolism in patients with Phlegm-stasis of IS and the coagulation function in patients with Phlegm-stasis of CHD
Zhao et al. (2018)	China	ICH	Stasis-heat	18	≤48 h	Lymphocytes	Microarray chips	Identified 4,744 differential genes: 2867↑, 1877↓
								The essence of Stasis-heat was related to coagulation and inflammatory pathology
Li et al. (2019)	China	ICH	Hyperactive liver-yang	15	NA	Fecal samples	16S rRNA	Decrease in relative abundance of prevotella and ackermann Myxobacteria
Wei et al. (2019)	China	PSCI	Blood-stasis obstructing the meridians; Fu-organ turbidness stagnation; Heat-toxin exuberance; Hyperactive liver-yang; Kidney-essence deficiency; Phlegm-turbidity obstructing the orifices; Qi-blood deficiency	190	NA	Leucocytes	PCR-RFLP	Methyl-tetrahydrofolate reductase gene G677T
								CT genotype may be related to Phlegm-turbidity obstructing the orifices and Blood-stasis obstructing the meridians, TT genotype may be related to Kidney-essence deficiency

ARMS-PCR, amplification refractory mutation system-polymerase chain reaction; CHD, coronary atherosclerotic heart disease; HDL, high density lipoprotein; ICH, intracerebral hemorrhage; IS, ischemic stroke; NA, not available; PBMCs, peripheral blood mononuclear cells; PCR-RFLP, polymerase chain reaction-restriction fragment length polymorphism; PSCI, post-stroke cognitive impairment; PVB, peripheral venous blood; TG, triglyceride; TCM, traditional Chinese medicine; UA, unstable angina.

**TABLE 2:** Summary of the main findings of the included transcriptomics studies.

Source	Sites	Disease	TCM syndromes	Sample size	Onset time	Specimen	Technology	Main findings
Zhao et al. (2018)	China	IS	Yang; Yin	22	≤6 h	Lymphocytes	Microarray chips	Yang related miRNAs: hsa-miR-93-5p, miR-320b, miR-320a, miR-128, and miR-181a-5p; Yin related miRNAs: hsa-miR-424-5p, miR-7-5p, miR-106b-5p, miR-19a-3p, and miR-301a-3p
Liu et al. (2019)	China	IS	Yang; Yin	30	NA	Serum	Microarray chips	Yang: identified 227 lncRNAs (73↑, 154↓), 54 mRNAs (21↑, 33↓), and 4 miRNAs; Yin: identified 394 lncRNAs (283↑, 111↓), 206 mRNAs (177↑, 29↓)
Liao et al. (2016)	China	IS; UA	Blood-stasis	15	≤48 h	PBMCs	Microarray chips	MiR-146b-5p, -199a-5p and 23 targeted mRNAs formed network-type biomarkers for Blood-stasis

(↑) upregulated; (↓) downregulated; IS, ischemic stroke; NA, not available; PBMCs, peripheral blood mononuclear cells; UA, unstable angina.

**TABLE 3:** Summary of the main findings of the included proteomics studies.

Source	Sites	Disease	TCM syndromes	Sample size	Onset time	Specimen	Technology	Main findings
Zeng et al. (2008)	China	IS	Liver-yang transforming into wind; Wind-stirring due to yin-deficiency	45	NA	Lymphocytes	2-DE; MALDI-TOF-MS	Identified 15 differential proteins, including capping protein, adenylyl cyclase-associated protein 1 and platelet thrombin sensitive protein-1
Xiong et al. (2011)	China	ICH; IS; CS; PD	Liver-yang transforming into wind; Hyperactive liver-yang; Wind-stirring due to yin-deficiency; Wind-stirring due to blood-deficiency	135	NA	PBMCs	2-DE; MALDI-TOF-MS	Thioredoxin-dependent peroxide reductase may be a specific marker protein of Liver yang transforming into wind
Wang et al. (2012)	China	IS	Liver-yang transforming into wind; Wind-stirring due to yin-deficiency	42	NA	Serum	2-DE; MALDI-TOF-MS	Liver-yang transforming into wind: 7 proteins↑ (ceruloplasmin, monocyte chemotaxis protein-1, c-reactive protein, etc.); Wind-stirring due to yin-deficiency: 5 proteins↑ (neuronspecific enolase, glycoprotein, signaling protein, etc.)
Li et al. (2014)	China	IS	Blood-stasis	27	≤72 h	Plasma	2-DE; MALDI-TOF-MS	Identified 6 differential proteins: 5↑ (haptoglobin, fibrinogen gamma chain, gamma-actin, SP40/40, vascular Rab-GAP/TBC-containing protein), 1↓ (TROVE domain family, member 2)
Zhao et al. (2008)	China	ICH	Liver-yang transforming into wind	20	NA	Lymphocytes	2-DE; MALDI-TOF-MS	Identified 8 differential proteins: 6↓ (alpha-enolase, apolipoprotein A-1, fibrinogen alpha chain, etc.),1↑ (hypothetical protein), 1 missing
Xiong et al. (2007)	China	HICH; HTN	HICH: liver-yang transforming into wind; HTN: hyperactive liver-yang	50	NA	Serum	2-DE; MALDI-TOF-MS	Identified 5 differential proteins, such as amyloid precursor protein, ceruloplasmin and vitamin D-binding protein
Xiao et al. (2008)	China	HICH; HTN	HICH: liver-yang transforming into wind; HTN: hyperactive liver-yang	45	NA	Lymphocytes	2-DE; MALDI-TOF-MS	Identified 16 differential proteins: 3↑ (gamma-actin, glutathione s-transferase omega-1, filamentous actin), 13↓ (zinc-binding protein, capping protein, thioredoxin-dependent peroxide reductase, etc.)
Chen et al. (2013)	China	ICH	Wind-stirring due to yin-deficiency	30	NA	Lymphocytes	2-DE; MALDI-TOF-MS	Identified 10 differential proteins: 5↑ (FilaminA, glucose-regulated protein, Zyxin protein, etc.), 4↓ (fibrinogen beta chain precursor, cofilin-1, hypothetical protein, glyceraldehyde-3-phosphate dehydrogenase), 1 missing
Yang et al. (2014)	China	ICH	Liver-yang transforming into wind	20	≤72 h	Lymphocytes	2-DE; MALDI-TOF-MS	Identified 3 differential proteins: actin, hypothetical protein and fibrinogen alpha chain precursor
Zhang et al. (2019)	China	ICH	Stasis-heat	8	≤48 h	Plasma	Tandem Mass Tag; UPLC-MS	Identified 7 differential proteins, such as ceruloplasmin, alpha-1B-glycoprotein, and carbonic anhydrase-1. Stasis-heat was related to inflammatory reaction and coagulation related to dysfunction

(↑) upregulated; (↓) downregulated; CS, cervical spondylosis; HICH, hypertensive intracerebral hemorrhage; HTN, hypertension; ICH, intracerebral hemorrhage; IS, ischemic stroke; MALDI-TOF-MS, matrix-assisted laser desorption/ionization time-of-flight mass spectrometry; NA, not available; PBMCs, peripheral blood mononuclear cells; PD, Parkinson’s disease; 2-DE, two-dimensional gel electrophoresis; UPLC-MS, ultra-performance liquid chromatography-mass spectrometry.

**TABLE 4:** Summary of the main findings of the included metabolomics studies.

Source	Sites	Disease	TCM syndromes	Sample size	Onset time	Specimen	Technology	Main findings
Cha et al. (2013)	Korean	IS	Phlegm-dampness	141	≤72 h	Plasma	UHPLC-MS	Plasma lysophosphatidylcholines with polyunsaturated fatty acid groups were associated with Phlegm-dampness
Cha et al. (2015)	Korean	IS	Blood-stasis	62	≤30 days	Plasma	UPLC-Q-TOF-MS	7 metabolites were related to Blood-stasis, including acyl-carnitines, creatinine and kynureninem
Rong and Li, (2020)	China	IS	Phlegm-dampness	31	≤72 h	Serum	NMR	30 metabolites were potential biomarkers for Phlegm-dampness, including 1-methyl histidine, alanine and acetic acid
Li et al. (2022)	China	IS	Yang-deficiency	20	>4 w	Serum	GC-TOF-MS	27 metabolites were related to Yang-deficiency, including glycine, fumaric acid, and aspartic acid
Yang et al. (2019)	China	ICH	Stasis-heat	120	≤48 h	Plasma	UPLC-MS	Cortisone 21 acetate, methyl acetate and triglyceride were potential biomarkers for Stasis-heat

GC-TOF-MS, gas chromatography-time of flight-mass spectrometry; ICH, intracerebral hemorrhage; IS, ischemic stroke; NMR, nuclear magnetic resonance; UHPLC-MS, ultra-high performance liquid chromatography-mass spectrometry; UPLC-MS, ultra-performance liquid chromatography-mass spectrometry; UPLC-Q-TOF-MS, ultra-performance liquid chromatography-quadrupole-time offlight-mass spectrometry.

**TABLE 5:** Summary of TCM syndromes and omics methods in studies included.

TCM syndromes of stroke	Number of genomics studies		Number of transcriptomics studies	Number of proteomics studies	Number of metabonomics studies
	SNP	DEGs			
IS					
Blood-stasis	5	0	1	1	1
Fire-heat	2	0	0	0	0
Hyperactive liver-yang	2	0	0	0	0
Liver-yang transforming into wind	0	0	0	2	0
Phlegm	4	0	0	0	0
Phlegm-dampness	0	0	0	0	2
Phlegm-heat and fu-organ excess	2	0	0	0	0
Phlegm-stasis	6	0	0	0	0
Qi-deficiency	3	0	0	0	0
Qi-deficiency and blood-stasis	9	0	0	0	0
Wind	3	0	0	0	0
Wind-phlegm and blood-stasis	1	0	0	0	0
Wind-phlegm and fire-hyperactivity	1	0	0	0	0
Wind-phlegm obstructing the meridians	2	0	0	0	0
Wind-phlegm stagnation	7	0	0	0	0
Wind-stirring due to yin-deficiency	3	0	0	2	0
Yang	0	0	2	0	0
Yang-deficiency	0	0	0	0	1
Yin	0	0	2	0	0
ICH					
Hyperactive liver-yang	0	0	1	0	0
Liver-yang transforming into wind	0	0	0	4	0
Stasis-heat	0	0	1	1	1
Wind-stirring due to yin-deficiency	0	0	0	2	0
PSCI					
Blood-stasis obstructing the meridians	1	0	0	0	0
Fu-organ turbidness stagnation	1	0	0	0	0
Hyperactive liver-yang	1	0	0	0	0
Heat-toxin exuberance	1	0	0	0	0
Kidney-essence deficiency	1	0	0	0	0
Phlegm-turbidity obstructing the orifices	1	0	0	0	0
Qi-blood deficiency	1	0	0	0	0

DEGs, differential expression genes; ICH, intracerebral hemorrhage; IS, ischemic stroke; PSCI, post-stroke cognitive impairment; SNP, single nucleotide polymorphism; TCM, traditional Chinese medicine.

### 3.1 Genomic studies on traditional Chinese medicine syndromes of stroke

Human genomics, the study of the structure, function, and interactions of all genes in the human genome, are widely used to elucidate the scientific basis of TCM syndrome differentiation (Lazaridis and Petersen, 2005). In this section, 25 studies (Huang et al., 2008; Jia et al., 2008; Hu et al., 2009; Shang et al., 2012; Xie et al., 2013; Shen et al., 2015; Gu et al., 2016a; Gu et al., 2016b; Gu et al., 2016c; Gu et al., 2016d; Huo and Tan, 2016; Wang et al., 2016; Gu et al., 2017; Zhao et al., 2018; Gu et al., 2019a; Gu et al., 2019b; Gu et al., 2019c; Gu et al., 2019d; Li et al., 2019; Wei et al., 2019; Zhu et al., 2019; Gu et al., 2020a; Gu et al., 2020b; Zhang et al., 2020; Gu et al., 2021), containing IS, ICH and post-stroke cognitive impairment, mainly covered research on single nucleotide polymorphisms (SNP) and differentially expressed genes (DEGs) (Table 1).

### 3.1.1 Ischemic stroke

Genomics studies on TCM syndromes of IS have focused on SNP, which are the most common cause of DNA sequence polymorphism (Griffin and Smith, 2000). To some extent, SNP has few similarities with the “Innate Endowmentt” of TCM, which may affect an individual’s susceptibility to a specific TCM syndrome (Yang et al., 2021). All included studies collected peripheral venous blood at baseline to extract DNA, and then carried out primer design, synthesis, and polymerase chain reaction (PCR) amplification. Finally, specific technology was used for SNP genotyping.

Most studies found that some gene polymorphisms may be pivotal in the occurrence of Wind-phlegm stagnation syndrome of IS, including the platelet glycoprotein Iba (GP Iba) gene *VNTR*, toll-like receptor 5 gene *rs5744174*, toll-like receptor 7 gene *rs2897827*, and tumor necrosis factor receptor-associated factor 6 (*TRAF6*) gene *rs5030416* (Shang et al., 2012; 2016; Gu et al., 2016c; Wang et al., 2016; Gu et al., 2017). Likewise, some gene polymorphisms, such as the GP Iba gene *VNTR* and *CYP2C19* gene may be associated with Qi-deficiency and blood-stasis syndrome in IS (Shang et al., 2012; Zhang et al., 2020). Additionally, methylenetetrahydrofolate reductase *C677T* and selenocysteine insertion sequence binding protein 2 gene *rs3211703* may be related to the pathogenesis of Blood-stasis syndrome of IS (Hu et al., 2009).

In addition to elucidating the scientific basis of TCM syndromes, researchers also revealed the relationships between gene polymorphisms and bodily functions in IS patients with specific TCM syndromes, including coagulation function, immunity, and blood lipid metabolism. The findings included the linkage between fibrinogen β-148C/T and coagulation function in patients with Wind-phlegm obstructing the meridians syndrome (Huo and Tan, 2016), between *TRAF6* gene *rs5030411* and inflammatory reaction in patients with Wind-phlegm stasis syndrome (Gu et al., 2017), and between myeloid differentiation factor 88 gene *rs7744*, histone deacetylase 9 gene *rs2107595* and coagulation function in patients with Wind-phlegm stagnation syndrome (Gu et al., 2016a; Zhu et al., 2019).

In particular, three studies focused on different diseases with the same TCM syndrome, including Phlegm-stasis syndrome in patients with IS or coronary atherosclerotic heart disease (CHD). The results showed that coagulation factor X gene *rs3093261*, *EP300* gene *rs20551*, and kinase insert domain receptor genes *rs2305948* and *rs2239702* may be related to the coagulation function in patients with Phlegm-stasis syndrome of IS and CHD, which indicated that different diseases with the same TCM syndrome may have the same biological basis (Gu et al., 2019b; Gu et al., 2019c; Gu et al., 2019d).

### 3.1.2 Intracerebral hemorrhage

Stasis-heat syndrome and Hyperactive liver-yang syndrome were the main syndromes of ICH in the two included studies, wherein the biological basis of TCM syndromes was investigated using DEGs. On the one hand, a total of 4,744 DEGs were identified in patients with Stasis-heat syndrome by using microarray chips to analyze lymphocytes (Zhao et al., 2018). Among these DEGs, few were further verified by real-time quantitative PCR, indicating that the Stasis-heat syndrome was intrinsically associated with coagulation and inflammatory pathology. On the other hand, fecal samples from patients were tested using the Illumina MiSeq platform, indicating that the Hyperactive liver-yang syndrome was related to the structural disorder of intestinal flora, with a reduced relative abundance of *Prevotella* and *Ackermann myxobacteria* (Li et al., 2019).

### 3.1.3 Post-stroke cognitive impairment

One study investigated the relationship between gene polymorphisms and TCM syndromes of post-stroke cognitive impairment using PCR-restriction fragment length polymorphism technology (Wei et al., 2019). Kidney-essence deficiency syndrome, Phlegm-turbidity obstructing the orifice syndrome, and Blood-stasis obstructing the meridians syndrome, all had a significant correlation with *MTHFR* gene *C677T* locus polymorphisms. Further correlation analysis showed that the former TCM syndrome was associated with the TT genotype, and the two latter TCM syndromes were associated with the CT genotype.

### 3.2 Transcriptomic studies on traditional Chinese medicine syndromes of stroke

Transcriptomics is the study of all transcripts in cells using microarray or RNA sequencing, including coding and non-coding RNAs, which is beneficial for revealing the intrinsic regulatory mechanisms of TCM syndromes (Manzoni et al., 2018; Montaner et al., 2020). Microarray technology is limited by the amount of RNA, quantification of transcript levels, and sequence information, however, it can still reveal the biological basis of TCM syndromes to a certain extent (Mutz et al., 2013). Microarray chips were used to investigate the transcriptomic characteristics of TCM syndromes of IS in the three included studies (Table 2) (Liao et al., 2016; Liu et al., 2019; Zhao et al., 2019).

Liu et al. (2019) and Zhao et al. (2019) focused on two TCM syndromes (Yin and Yang) of IS. The former found that there were some differences in the expression profiles of lncRNA, mRNA, and miRNA between the Yin syndrome and Yang syndrome. Further enrichment analysis revealed that the phenotypic differences between the Yin syndrome and Yang syndrome may be caused by blood pressure regulation, adrenergic receptor regulation, the renin-angiotensin system, and other pathways. The latter also identified some differentially expressed miRNAs; further enrichment analysis indicated that the key regulatory miRNAs, genes and pathways in Yang syndrome were hsa-miR-93-5p and -320b, enabled homologs, metabolic pathways, and mitogen-activated protein kinase signaling pathways, respectively, while those in Yin syndrome were hsa-miR-424-5p and -106b-5p, CNOT4, hepatitis B and pathways in cancer, respectively. Liao et al. (2016) found that 401 mRNAs and 11 miRNAs were differentially expressed in two conditions (IS and unstable angina) with the same TCM syndrome (Blood-stasis). Further bioinformatics analysis with validation by real-time quantitative PCR in an independent cohort demonstrated that miR-146b-5p, -199a-5p and 23 targeted mRNAs formed network-type biomarkers for the Blood-stasis syndrome.

### 3.3 Proteomic studies on traditional Chinese medicine syndromes of stroke

The aim of proteomics in modern biology is to understand the expression, function, and regulation of the entire set of proteins encoded by an organism, the information of which will be invaluable for understanding how complex biological processes occur at a molecular level (Zhu et al., 2003). Proteomics research is conducive to exploring the microscopic material basis of TCM syndromes at the surface level (Zhou et al., 2012; Yang et al., 2021). At present, proteomic studies on TCM syndromes of stroke have mainly focused on exploring differential proteins (Table 3) (Xiong et al., 2007; Xiao et al., 2008; Zeng et al., 2008; Zhao et al., 2008; Xiong et al., 2011; Wang et al., 2012; Chen et al., 2013; Li et al., 2014; Yang et al., 2014; Zhang et al., 2019).

### 3.3.1 Ischemic stroke

A total of three studies were conducted by using two-dimensional gel electrophoresis (2DE) combined with matrix-assisted laser desorption/ionization time-of-flight mass spectrometry (MALDI-TOF-MS) analyses, in which Blood-stasis syndrome and Liver-yang transforming into wind syndrome of IS were investigated (Zeng et al., 2008; Wang et al., 2012; Li et al., 2014). Six differentially expressed proteins were identified in Blood-stasis syndrome of IS, of which one protein (TROVE domain family, member 2) was downregulated, while the others, such as haptoglobin, fibrinogen gamma chain, and gamma-actin, were upregulated (Li et al., 2014). Interestingly, Zeng et al. (2008) and Wang et al. (2012) compared the Liver-yang transforming into wind syndrome with Wind-stirring due to yin-deficiency syndrome of IS, but their results were different. The former identified 15 differentially expressed proteins, including capping protein, adenylyl cyclase-associated protein 1, and platelet thrombin sensitive protein-1. The latter identified seven upregulated proteins in the Liver-yang transforming into wind syndrome, including ceruloplasmin, monocyte chemotaxis protein-1, and c-reactive protein, and five upregulated proteins in Wind-stirring due to yin-deficiency syndrome, including neuron-specific enolase, glycoprotein, and signaling proteins.

In addition, Xiong et al. (2011) tried to reveal the biological mechanism of Liver-yang transforming into wind syndrome in four different diseases: including ICH, IS, Parkinson’s disease, and cervical spondylosis. Through the application of 2DE combined with MALDI-TOF-MS technology and further comparative analysis, the results demonstrated that thioredoxin-dependent peroxide reductase may be a common marker protein of Liver-yang transforming into wind syndrome in multiple diseases.

### 3.3.2 Intracerebral hemorrhage

Similar to proteomic studies on TCM syndromes of IS, there were five other studies using 2DE combined with MALDI-TOF-MS technology to carry out related TCM syndromes of ICH, including Wind-stirring due to yin-deficiency syndrome and Live-yang transforming wind syndrome (Xiong et al., 2007; Xiao et al., 2008; Zhao et al., 2008; Chen et al., 2013; Yang et al., 2014). Ten differential proteins were finally identified in Wind-stirring due to yin-deficiency syndrome, five of which were upregulated, including FilaminA, Zyxin protein, and glucose-regulated protein, while four were downregulated, including fibrinogen beta chain precursor, cofilin-1, and hypothetical protein (Chen et al., 2013). Eight differential proteins were identified in the Live-yang transforming wind syndrome, among which the hypothetical protein was upregulated, and six were downregulated, including alpha-enolase, apolipoprotein A-1, and fibrinogen alpha chain (Zhao et al., 2008). Moreover, two studies also focused on Liver-Yang transforming wind syndrome; however, they only concentrated on ICH caused by hypertension (Xiong et al., 2007; Xiao et al., 2008). In comparison with hypertensive patients with Hyperactive liver-yang syndrome and healthy individuals, one study identified five differentially expressed, including amyloid precursor protein, ceruloplasmin, and vitamin D-binding protein (Xiong et al., 2007); the other identified 16 differential proteins, three of which were upregulated, such as gamma-actin, glutathione s-transferase omega-1, and filamentous actin, while 13 were downregulated, such as zinc-binding protein, capping protein, and thioredoxin-dependent peroxide reductase (Xiao et al., 2008).

Of note, one study included 10 patients with Liver-yang transforming into wind syndrome of ICH within 3 days of onset, and thereafter observed patients whose TCM syndrome changed to Wind-stirring due to yin-deficiency syndrome within 3 months (Yang et al., 2014). Through comparative analysis before and after, actin, hypothetical protein, and fibrinogen alpha chain precursor may be closely related to the dynamic evolution of these two TCM syndromes. Furthermore, through Tandem Mass Tag combined with ultra-performance liquid chromatography-mass spectrometry (UPLC-MS), Zhang et al. (2019) finally identified seven differential proteins, such as ceruloplasmin, alpha-1B-glycoprotein, and carbonic anhydrase-1, which proved that the basis of Stasis-heat syndrome may be related to inflammatory reactions and coagulation related to dysfunctions.

### 3.4 Metabolomic studies on traditional Chinese medicine syndromes of stroke

Metabolomics is defined as the comprehensive analysis in which all the metabolites of a biological system are identified and quantified (Lindon and Nicholson, 2008). Unlike genes and proteins, metabolites serve as direct signatures of biochemical activity and are therefore, easier to correlate with phenotypes (Ussher et al., 2016). Nuclear magnetic resonance (NMR) spectroscopy and mass spectrometry combined with chromatography are the main primary analytical methods. In this section, five studies were included, four of which focused on TCM syndromes of IS (Cha et al., 2013; Cha et al., 2015; Rong and Li, 2020; Li et al., 2022), whereas the rest focused on TCM syndromes of ICH (Table 4) (Yang et al., 2019).

### 3.4.1 Ischemic stroke

Metabolomic studies on the TCM syndromes of IS carried out by different groups have helped elucidate the biological basis of TCM syndromes. Phlegm-dampness syndrome, Yang-deficiency syndrome, and Blood-stasis syndrome were the main syndromes investigated in the four included studies. There were two studies on Phlegm-dampness syndrome using different metabolomics technologies (Cha et al., 2013; Rong and Li, 2020). The former used NMR and found 30 different metabolites, including 1-methylhistidine, alanine, and acetic acid, which were potential biomarkers for the Phlegm-dampness syndrome of IS (Cha et al., 2013). The latter used UPLC-MS and found that the levels of lysophosphatidylcholine (18:2) and lysophosphatidylcholine (20:3) in the Phlegm dampness syndrome were low, suggesting that the variation in plasma lipid profiles may serve as a potential biomarker for its diagnosis (Rong and Li, 2020). In addition, Seven differential metabolites, including acyl-carnitines, creatinine, and kynureninem, were identified to be associated with Blood-stasis syndrome by using ultra-high performance liquid chromatography-quadrupole-time of flight-mass spectrometry (Yang et al., 2019). In addition, the gas chromatography-time of flight-mass spectrometry method was applied in the study of Yang-deficiency syndrome, with 27 metabolites as potential markers (Li et al., 2022).

### 3.4.2 Intracerebral hemorrhage

Metabolomics has also been used to gain insights into the biological basis of TCM syndromes in ICH. Yang et al. (2019) investigated the metabolomic characteristics of the Stasis-heat syndrome of ICH by analyzing peripheral blood using UPLC-MS. The results showed that the pathogenesis of Stasis-heat syndrome of ICH was related to excess oxidative stress, inflammatory response, vascular sclerosis, and apoptosis. Moreover, the obtained cortisone 21 acetate, methyl acetate, and triglycerides could be used as potential biomarkers for the Stasis-heat syndrome of ICH.

## 4 Discussion

This systematic review provided a summary of the application of multi-omics approaches to reveal the biological basis of TCM syndromes in stroke. Omics data have prompted elucidation of the remarkable complexity of TCM syndromes. The current results demonstrated that some gene polymorphisms, differential lncRNAs, mRNAs, miRNAs, proteins, and metabolites may all be associated with TCM syndromes of stroke. Of note, some studies conducted a preliminary exploration of different diseases with the same TCM syndrome. The results showed that thioredoxin-dependent peroxidase reductase may be a common marker protein of Liver-yang transforming into wind syndrome, and the network formed by mir-146b-5p, -199a-5p, and 23 targeted mRNAs may be the biomarker of Blood-stasis syndrome. These results brought us closer to deciphering the biological basis of TCM syndromes in stroke, and even uncovered some potential biomarkers, especially for Liver-yang transforming into wind syndrome and Blood-stasis syndrome. With further validation, these findings may be applied to the objective diagnosis of TCM syndromes in future clinical practice and may also be potential targets of Chinese herbal medicine, which will facilitate the discovery of new therapeutic drugs. However, these results should be interpreted with caution because of the moderate quality of the included studies.

In addition to the omics methods used in the included studies, there are also epigenomics, glycomics, and lipidomics, which generate a large amount of information at the sample level but neglect the characteristics within individual cells concurrently. As new omics technologies continue to emerge, single-cell omics technologies, including single-cell genomes, transcriptomes, epigenomes, proteomes, and metabolomes, may be able to address cellular level heterogeneities and further discover new diagnostic and therapeutic targets (Islam et al., 2020). Currently, it has been used in the exploration of TCM syndromes. For example, Lu et al. (2021) used single-cell RNA sequencing technology to explore the significance of tumor heterogeneity in the classification of TCM syndromes of colorectal cancer and finally found that Excess syndrome and Deficiency syndrome may be related to tumor heterogeneity.

Notably, none of these included studies performed multi-omics integrated analysis. These included studies only stayed at a single omics level, ignoring the crosstalk between different molecular entities at different omics levels, which may have led to the omission of biologically relevant information. We did not perform an integrated analysis in this review due to significant heterogeneity among participants from different studies as well as the limited data from these included original articles. The main goal of omics research is not merely to find identifiable differences at the single-omics level but also to decipher the complexity of TCM syndromes at multiple layers at the molecular-level. Consequently, the integration of multi-omics data is becoming crucial for in-depth understanding of the biological basis of TCM syndromes in stroke, which ultimately leads to the discovery of biomarkers and novel therapeutic drugs, and promotes the modernization of TCM and personalized medicine of TCM in stroke (Gu and Chen, 2014; Guo et al., 2020). Future omics research on TCM syndromes should focus on the multi-omics levels and get the utmost out of high-precision algorithms. The best strategy to conduct integrated multi-omics analysis would rely on proteomics, genomics, transcriptomics, and metabolomics data which are acquired simultaneously from participants with low heterogeneity.

Fortunately, it was encouraging to see some promising findings recently, in which Li et al. (2007) established a network research paradigm integrating high-precision computational prediction, multi-omics data and experimental validation. Using this network strategy, we successfully discovered the molecular network characteristics of Spleen-deficiency syndrome at multiple molecular levels, and revealed that this TCM syndrome was closely related to insufficient immune response, including decreased macrophage activity and decreased lymphocyte proliferation (Wang et al., 2020).

Despite promising findings and advances in this field, multi-omics research related to TCM syndromes of stroke still needs to be improved in future studies. First, a recognized and consistent standard for TCM syndrome diagnosis or identification is fundamental for further uncovering the biological mechanism of TCM syndromes. Second, the control group should be set reasonably and strictly matched with the group of interest TCM syndrome to eliminate confounding factors. Third, to ensure data quality, strict standard operating procedures (SOP) should be set for sample collection, storage, processing and acquisition, such as SOP for the collection, storage, and transportation of fecal samples that conform to the unique features of TCM for fecal samples (Su et al., 2022). Fourth, external validation of biomarkers for potential TCM syndromes should be performed before incorporating them into clinical practice.

## 5 Conclusion

In facing the great challenge of research on TCM syndromes, multi-omics technologies combined with high-precision computational algorithms have served as powerful tools to investigate the complexity of TCM syndromes and may hold the promise of promoting the modernization of TCM as well as personalized medicine of TCM in stroke.

## Data Availability

The original contributions presented in the study are included in the article/Supplementary Material, further inquiries can be directed to the corresponding authors.

## Abbreviations

CHD, coronary atherosclerotic heart disease; CNKI, China National Knowledge Infrastructure; DE, dimensional gel electrophoresis; DEGs, differentially expressed genes; GP, glycoprotein; ICH, intracerebral hemorrhage; IS, ischemic stroke; MALDI-TOF-MS, matrix-assisted laser desorption/ionization time-of-flight mass spectrometry; NMR, nuclear magnetic resonance; PCR, polymerase chain reaction; SinoMed, China Biology Medicine Database; SNP, single nucleotide polymorphism; SOP, strict standard operating procedure; TCM, traditional Chinese medicine; TRAF6, tumor necrosis factor receptor-associated factor 6; UPLC-MS, ultra-performance liquid chromatography-mass spectrometry; VIP, Chinese Science and Technology Periodical Database; WOSCC, Web of Science Core Collection.
