# Supplementary material for: Multi-omics approaches for deciphering the complexity of traditional Chinese medicine syndromes in stroke: A systematic review
Source: Front Pharmacol. 2022 Sep 6;13:980650. doi: 10.3389/fphar.2022.980650 (PMC9489218; doi:10.3389/fphar.2022.980650)
Supplement: Supplementary file 1 [file Table1.DOCX]

**Supplementary Table 1** Search Strategy Example: PubMed search

| **No** | **Search items** |
| --- | --- |
| 1 | Genomics |
| 2 | Comparative Genomics |
| 3 | Comparative Genomic |
| 4 | Genomic, Comparative |
| 5 | Genomics, Comparative |
| 6 | Metabolomics |
| 7 | Metabolomic |
| 8 | Metabonomics |
| 9 | Metabonomic |
| 10 | Epigenomics |
| 11 | Epigenomic |
| 12 | Epigenetics |
| 13 | Epigenetic |
| 14 | Glycomics |
| 15 | Glycobiology |
| 16 | Proteomics |
| 17 | Lipidomics |
| 18 | Lipidomic |
| 19 | Lipidome |
| 20 | Lipidomes |
| 21 | Transcriptomics |
| 22 | Omics |
| 23 | Multi-omics |
| **24** | **1 OR 2-23** |
| 25 | Stroke |
| 26 | Strokes |
| 27 | Cerebrovascular Accident |
| 28 | Cerebrovascular Accidents |
| 29 | CVA |
| 30 | CVAs |
| 31 | Cerebrovascular Apoplexy |
| 32 | Apoplexy, Cerebrovascula |
| 33 | Vascular Accident, Brain |
| 34 | Brain Vascular Accident |
| 35 | Brain Vascular Accidents |
| 36 | Vascular Accidents, Brain |
| 37 | Cerebrovascular Stroke |
| 38 | Cerebrovascular Strokes |
| 39 | Stroke, Cerebrovascular |
| 40 | Strokes, Cerebrovascular |
| 41 | Apoplexy |
| 42 | Cerebral Stroke |
| 43 | Cerebral Strokes |
| 44 | Stroke, Cerebral |
| 45 | Strokes, Cerebral |
| 46 | Stroke, Acute |
| 47 | Acute Stroke |
| 48 | Acute Strokes |
| 49 | Strokes, Acute |
| 50 | Cerebrovascular Accident, Acute |
| 51 | Acute Cerebrovascular Accident |
| 52 | Acute Cerebrovascular Accidents |
| 53 | Cerebrovascular Accidents, Acute |
| 54 | Cerebral Hemorrhage |
| 55 | Cerebral Hemorrhages |
| 56 | Brain Hemorrhage |
| 57 | Brain Hemorrhages |
| 58 | Intracerebral Hemorrhage |
| 59 | Intracerebral Hemorrhages |
| 60 | Cerebrum Hemorrhage |

| 61 | Cerebrum Hemorrhages |
| --- | --- |
| 62 | Brain Ischemia |
| 63 | Brain Ischemias |
| 64 | Cerebral Ischemia |
| 65 | Cerebral Ischemias |
| 66 | Brain Infarction |
| 67 | Brain Infarctions |
| 68 | Cerebral Infarction |
| 69 | Cerebral Infarctions |
| 70 | Ischemic Stroke |
| 71 | Hemorrhagic Stroke |
| **72** | **25 OR 26-71** |
| 73 | Medicine, Chinese Traditional |
| 74 | Traditional Chinese Medicine |
| 75 | Chung I Hsueh |
| 76 | Hsueh, Chung I |
| 77 | Traditional Medicine, Chinese |
| 78 | Zhong Yi Xue |
| 79 | Chinese Traditional Medicine |
| 80 | Chinese Medicine, Traditional |
| 81 | Traditional Tongue Diagnosis |
| 82 | Tongue Diagnoses, Traditional |
| 83 | Tongue Diagnosis, Traditional |
| 84 | Traditional Tongue Diagnoses |
| 85 | Traditional Tongue Assessment |
| 86 | Tongue Assessment, Traditional[ |
| 87 | Traditional Tongue Assessments |
| 88 | Drugs, Chinese Herbal |
| 89 | Chinese Drugs, Plant |
| 90 | Chinese Herbal Drugs |
| 91 | Herbal Drugs, Chinese |
| 92 | Plant Extracts, Chinese |
| 93 | Chinese Plant Extracts |
| 94 | Extracts, Chinese Plant |
| 95 | Phytotherapy |
| 96 | Herbal Therapy |
| 97 | Herb Therapy |
| 98 | ZHENG |
| 99 | Chinese medicine ZHENG |
| 100 | Chinese medicine syndrome |
| 101 | Syndrome differentiation |
| **109** | **73 OR 74-101** |
| **110** | **24 AND 72 AND 103** |
