# Supplementary material for: Multi-omics approaches for deciphering the complexity of traditional Chinese medicine syndromes in stroke: A systematic review
Source: Front Pharmacol. 2022 Sep 6;13:980650. doi: 10.3389/fphar.2022.980650 (PMC9489218; doi:10.3389/fphar.2022.980650)
Supplement: Supplementary file 2 [file Table2.DOCX]

**Supplementary Table 2** Evaluation of methodological quality of included studies.

| **Study** | **Define the source of informa-tion** | **List inclusion and exclusion criteria for exposed and unexposed subjects (cases and controls) or refer to previous publications** | **Indicate time period used for identifying patients** | **Indicate whether or not subjects were consecutive if not population-based** | **Indicate if evaluators of subjective components of study were masked to other aspects of the status of the participants** | **Describe any assessments undertaken for quality assurance purposes** | **Explain any patient exclusions from analysis** | **Describe how confounding was assessed and/or controlled** | **If applicable, explain how missing data were handled in the analysis** | **Summarize patient response rates and completeness of data collection** | **Clarify what follow-up, if any, was expected and the percentage of patients for which incomplete data or follow-up was obtained** | **Total score**  **（No or Unclear = 0, Yes = 1）** |
| --- | --- | --- | --- | --- | --- | --- | --- | --- | --- | --- | --- | --- |
| Jia et al,  2008 | Yes | Yes | Yes | Unclear | Unclear | No | Yes | Unclear | Yes | Yes | Unclear | 6 |
| Huang et al,  2008 | Yes | No | No | Unclear | Unclear | No | Yes | Unclear | Yes | Yes | Unclear | 4 |
| Hu et al,  2009 | Yes | Yes | No | Unclear | Unclear | No | Yes | Unclear | Yes | Yes | Unclear | 5 |
| Shang et al,  2012 | Yes | Yes | No | Unclear | Unclear | No | Yes | Unclear | Yes | Yes | Unclear | 5 |
| Xie et al,  2013 | Yes | Yes | No | Unclear | Unclear | No | Yes | Unclear | Yes | Yes | Unclear | 5 |
| Shen et al,  2015 | Yes | Yes | No | Unclear | Unclear | No | Yes | Unclear | Yes | Yes | Unclear | 5 |
| Huo et al,  2016 | Yes | Yes | Yes | Unclear | Unclear | No | Yes | Yes | Yes | Yes | Unclear | 6 |
| Wang et al,  2016 | Yes | Yes | No | Unclear | Unclear | No | Yes | Unclear | Yes | Yes | Unclear | 5 |
| Gu et al,  2016a | Yes | Yes | No | Unclear | Unclear | No | Yes | Unclear | Yes | Yes | Unclear | 5 |
| Gu et al,  2016b | Yes | Yes | Yes | Unclear | Unclear | No | Yes | Unclear | Yes | Yes | Unclear | 6 |
| Gu et al,  2016c | Yes | Yes | No | Unclear | Unclear | No | Yes | Unclear | Yes | Yes | Unclear | 5 |
| Gu et al,  2016d | Yes | Yes | No | Unclear | Unclear | No | Yes | Unclear | Yes | Yes | Unclear | 5 |
| Gu et al,  2017 | Yes | Yes | No | Unclear | Unclear | No | Yes | Unclear | Yes | Yes | Unclear | 5 |
| Zhu et al,  2019 | Yes | Yes | No | Yes | Unclear | No | Yes | Unclear | Yes | Yes | Unclear | 5 |
| Gu et al,  2019a | Yes | Yes | No | Unclear | Unclear | No | Yes | Unclear | Yes | Yes | Unclear | 5 |
| Gu et al,  2020a | Yes | Yes | Yes | Unclear | Unclear | No | Yes | Unclear | Yes | Yes | Unclear | 6 |
| Zhang et al,  2020 | Yes | Yes | No | Unclear | Unclear | No | Yes | Unclear | Yes | Yes | Unclear | 5 |
| Gu et al,  2021 | Yes | Yes | Yes | Unclear | Unclear | No | Yes | Unclear | Yes | Yes | Unclear | 6 |
| Gu et al,  2019b | Yes | Yes | No | Unclear | Unclear | No | Yes | Unclear | Yes | Yes | Unclear | 5 |
| Gu et al,  2019c | Yes | Yes | No | Unclear | Unclear | No | Yes | Unclear | Yes | Yes | Unclear | 5 |
| Gu et al,  2019d | Yes | Yes | No | Unclear | Unclear | No | Yes | Unclear | Yes | Yes | Unclear | 5 |
| Gu et al,  2020b | Yes | Yes | No | Unclear | Unclear | No | Yes | Unclear | Yes | Yes | Unclear | 5 |
| Zhao et al,  2018 | Yes | Yes | Yes | Unclear | Unclear | No | Yes | Unclear | Yes | Yes | Unclear | 6 |
| Li et al,  2019 | Yes | Yes | No | Unclear | Unclear | No | Yes | Unclear | Yes | Yes | Unclear | 5 |
| Wei et al,  2019 | Yes | Yes | No | Unclear | Unclear | No | Yes | Yes | Yes | Yes | Unclear | 6 |
| Zhao et al,  2018 | Yes | Yes | Yes | Unclear | Unclear | No | Yes | Yes | Yes | Yes | Unclear | 7 |
| Liu et al,  2019 | Yes | Yes | No | Unclear | Unclear | No | Yes | Yes | Yes | Yes | Unclear | 6 |
| Liao et al,  2016 | Yes | Yes | Yes | Unclear | Unclear | Yes | Yes | Unclear | Yes | Yes | Unclear | 7 |
| Zeng et al,  2008 | Yes | Yes | No | Unclear | Unclear | No | Yes | Unclear | Yes | Yes | Unclear | 5 |
| Xiong et al,  2011 | Yes | Yes | No | Unclear | Unclear | No | Yes | Unclear | Yes | Yes | Unclear | 5 |
| Wang et al,  2012 | Yes | Yes | No | Unclear | Unclear | No | Yes | Unclear | Yes | Yes | Unclear | 5 |
| Li et al,  2014 | Yes | Yes | Yes | Unclear | Unclear | No | Yes | Unclear | Yes | Yes | Unclear | 6 |
| Zhao et al,  2008 | Yes | Yes | No | Unclear | Unclear | No | Yes | Unclear | Yes | Yes | Unclear | 5 |
| Xiong et al,  2007 | Yes | No | No | Unclear | Unclear | No | Yes | Unclear | Yes | Yes | Unclear | 4 |
| Xiao et al,  2008 | Yes | No | No | Unclear | Unclear | No | Yes | Unclear | Yes | Yes | Unclear | 4 |
| Chen et al,  2013 | Yes | Yes | No | Unclear | Unclear | No | Yes | Unclear | Yes | Yes | Unclear | 5 |
| Yang et al,  2014 | Yes | Yes | Yes | Unclear | Unclear | No | Yes | Unclear | Yes | Yes | Yes | 7 |
| Zhang et al,  2019 | Yes | Yes | Yes | Unclear | Unclear | No | Yes | Yes | Yes | Yes | Unclear | 7 |
| Cha et al,  2013 | Yes | No | Yes | Unclear | Unclear | No | Yes | Unclear | Yes | Yes | Unclear | 5 |
| Cha et al,  2015 | Yes | Yes | Yes | Unclear | Unclear | No | Yes | Unclear | Yes | Yes | Unclear | 6 |
| Rong et al,  2020 | Yes | Yes | Yes | Unclear | Unclear | No | Yes | Unclear | Yes | Yes | Unlear | 6 |
| Li et al,  2022 | Yes | Yes | Yes | Unclear | Unclear | No | Yes | Unclear | Yes | Yes | Unclear | 6 |
| Yang et al,  2019 | Yes | Yes | Yes | Unclear | Unclear | No | Yes | Unclear | Yes | Yes | Unlear | 6 |
