# Supplementary material for: Multi-omics approaches for deciphering the complexity of traditional Chinese medicine syndromes in stroke: A systematic review
Source: Front Pharmacol. 2022 Sep 6;13:980650. doi: 10.3389/fphar.2022.980650 (PMC9489218; doi:10.3389/fphar.2022.980650)
Supplement: Supplementary file 3 [file Table3.DOCX]

**Supplementary Table 3** The list of 43 included studies

| 1. Cha, M. H., Jones, A. D., Ko, M. M., Zhang, C., and Lee, M. S. (2013). Metabolic Profiles Distinguish Non-Dampness-Phlegm and Dampness-Phlegm Patterns among Korean Patients with Acute Cerebral Infarction. Evid Based Complement Alternat Med*.* 2013, 517018. doi: 10.1155/2013/517018 2. Cha, M. H., Kim, M. J., Jung, J., Kim, J. H., Lee, M. S., and Kim, M. S. (2015). Metabolomic Analysis of Clinical Plasma from Cerebral Infarction Patients Presenting with Blood Stasis. Evid Based Complement Alternat Med*.* 2015, 453423. doi: 10.1155/2015/453423 3. Chen, J., Zhang, Y., Xiong, X., Liang, Q., and Zhao, Y. (2013). Proteomics study on the syndrome-effect relationship of Dading Fengzhu decoction for intracerebral hemorrhage patients with Wind-stirring due to yin-deficiency syndrome in recovery stage. Journal of Hunan University of traditional Chinese Medicine*.* 33(11), 57-62 4. Gu, L., Chen, Z., Li, M., Yan, Y., Liang, B., Yang, J., and Shen, T. (2019). Correlation between KDR gene rs2305948, rs2239702 polymorphism and coagulation function of ischemic stroke, coronary heart disease with Phlegm-stasis syndrome. Journal of Beijing University of Traditional Chinese Medicine*.* 32(4), 345-352 5. Gu, L., Chen, Z., Long, J., Zhu, L., and Su, L. (2021). Correlation between rs5744724 polymorphism of DNA polymerase kappa (Polk) gene and ischemic stroke with Qi-deficiency syndrome and Blood-stasis syndrome. Chinese Archives of Traditional Chinese Medicine*.* 39(6), 8-13 6. Gu, L., Chen, Z., Su, L., Yan, Y., Wu, G., Liang, B., Tan, J., and Tang, N. (2017). Association between TRAF6 gene polymorphisms and risk and inflammatory reactions of stroke with Wind-phlegm stagnation syndrome. Journal of Beijing University of Traditional Chinese Medicine*.* 40(12), 1030-1036 7. Gu, L., Chen, Z., Yan, Y., Long, J., Zhu, L., and Su, L. (2020). Selenocysteine insertion sequence binding protein 2 gene rs3211703 polymorphism is significantly associated with Blood-stasis syndrome and Qi-deficiency syndrome in ischemic stroke. Chinese Archives of Traditional Chinese Medicine*.* 38(6), 34-38+65 8. Gu, L., Gong, L., Huang, S., Li, J., Li, M., Li, T., and Su, L. (2019). Correlation analysis of STAT5A gene polymorphism rs319502 between coagulation function and inflammatory reaction in ischemic stroke of Phlegm-stasis syndrome. Liaoning Journal of Traditional Chinese Medicine*.* 46(11), 2241-2245 9. Gu, L., Huang, S., Li, M., Li, J., Li, T., Gong, L., and Shen, T. (2020). Association analyses between decorin gene rs7441 polymorphism and Phlegm-Stasis syndrome of ischemic stroke and coronary atherosclerotic heart disease. Chinese Archives of Traditional Chinese Medicine*.* 38(2), 130-134 10. Gu, L., Li, M., Li, J., Liang, B., Huang, S., and Su, L. (2019). Correlation between genetic polymorphisms of F10 gene and coagulation markers of Phlegm-stasis syndrome in ischemic stroke and Phlegm-stasis syndrome in coronary heart disease. Chinese Journal of Gerontology*.* 39(16), 3878-3884 11. Gu, L., Li, T., Li, M., Huang, S., Gong, L., Li, J., Jiang, H., and Liang, B. (2019). Relationship of EP300 gene rs20551 polymorphism with Phlegm-stasis syndrome and blood coagulation function in patients with ischemic stroke and coronary atherosclerotic heart disease. Chinese Journal of Geriatric Heart Brain and Vessel Diseases*.* 21(7), 720-724 12. Gu, L., Shen, T., Liang, B., Tan, J., Yan, Y., and Tang, N. (2016). GWAS-supported variant rs2107595 polymorphism influence the serum lipid metabolism of ischemic stroke patients with Wind-phlegm stagnation syndrome. China Journal of Traditional Chinese Medicine and Pharmacy*.* 31(11), 4484-4487 13. Gu, L., Wei, Q., Xie, J., Chen, Q., Liang, B., and Tang, N. (2016). Myeloid differentiation factor gene ( MYD88) polymorphism affects blood lipid metabolism in stroke with Phlegm-Stasis syndrome. Lishizhen Medicine and Materia Medica Research*.* 27(5), 1266-1288 14. Gu, L., Wu, Y., Liang, B., Tan, J., Chen, Q., Wei, Q., Shen, T., Jiang, H., and Chen, N. (2016). Association analysis of gene polymorphism of MAP2K4and MAPK1 and different traditional Chinese medicine syndromes and serum lipid levels in patients with ischemic stroke. J. Tradit. Chin. Med. 57(10), 864-869 15. Gu, L., Zhou, J., Chen, Q., Xie, J., Yan, Y., Liang, B., Tan, J., and Tang, N. (2016). TOLL-like receptor-7 gene rs2897827 polymorphism increase the risk on stroke with Wind-phlegm stagnation syndrome in Han nationality females. China Journal of Traditional Chinese Medicine and Pharmacy*.* 31(10), 4219-4222 16. Hu, M., Wang, Z., Li, T., Zhang, X., Xing, Z., Zhou, C., and Su, L. (2009). Study on MTHFR C677T polymorphism in ischemic stroke with Blood-stasis syndrome. Chinese Journal of Information on Traditional Chinese Medicine*.* 16(3), 16-18 17. Huang, B., Shao, W., Dong, M., Tu, J., and Wang, L. (2008). Association between traditional Chinese medicine syndromes in cerebral infarction and angiotensinogen gene M235T polymorphism. Medical Journal of Wuhan University*.* 29(4), 482-484+507 18. Huo, Q., and Tan, F. (2016). Study on traditional Chinese medicine syndromes of cerebral infarction and fibrinogen related gene polymorphism. Chinese Journal of Modern Drug Application*.* 10(12), 80-81 19. Jia, N., You, J., and Huang, P. (2008). Relationship between ACE gene polymorphism and initial traditional Chinese medicine syndrome of acute ischemic stroke. Liaoning Journal of traditional Chinese Medicine*.* 35(4), 481-483 20. Li, J., Chen, Z., Ma, Y., Wu, D., Hu, Y., and Li, J. (2022). Analysis of biomarkers of ischemic stroke with Yang-deficiency syndrome based on GC-TOF-MS combined with metabolomics. Hunan Journal of traditional Chinese Medicine*.* 38(1), 6-10 21. Li, S., Wang, F., Li, P., Zhao, H., Zhang, W., and Wang, Y. (2014). Preliminary study on proteomics of Blood-stasis syndrome in ischemic stroke. China Journal of Traditional Chinese Medicine and Pharmacy*.* 29(12), 3977-3980 22. Li, Y., Xu, H., Wang, J., Zheng, F., Deng, L., Li, J., Chen, L., Yuan, H., and Chen, X. (2019). Analysis of bacterial flora structure of patients with cerebral hemorrhage due to Hyperactive liver-yang syndrome by 16S rRNA gene sequencing technique. Chinese Journal of Experimental Traditional Medical Formulae*.* 25(8), 83-88 23. Liao, J., Liu, Y., and Wang, J. (2016). Identification of more objective biomarkers for Blood-Stasis syndrome Diagnosis. BMC Complem. Altern. M. 16(1), 371. doi: 10.1186/s12906-016-1349-9 24. Liu, W., Li, G., He, C., Qiao, L., Shen, X., Cheng, X., Cai, Y., and Huang, Y. (2019). Analysis of serum transcriptome characteristics of patients with Yin syndrome and Yang syndrome of acute ischemic stroke. Chinese Journal of Experimental Traditional Medical Formulae*.* 25(15), 122-130 25. Rong, L., and Li, Y. (2020). Study on biomarkers of Phlegm-dampness syndrome in ischemic stroke based on metabonomics. Journal of Guangzhou University of Traditional Chinese Medicine*.* 37(2), 195-200 26. Shang, Y., Bai, J., Shi, C., Li, T., Feng, Q., Zheng, H., and He, Y. (2012). Correlation between platelet membrane glycoprotein Iba gene polymorphism and traditional Chinese medicine syndromes of arteriosclerotic cerebral infarction. Lishizhen Medicine and Materia Medica Research*.* 23(8), 1994-1996 27. Shen, T., Gu, L., Chen, Q., Liang, B., and Yan, Y. (2015). Association study on rs2107595 polymorphism identified by GWAS and ischemic stroke of Qi-deficiency syndrome and Blood-stasis syndrome. Journal of Guangxi University of Traditional Chinese Medicine*.* 18(4), 5-8 28. Wang, L., Liang, Q., Chen, X., Yang, L., Tang, K., Liu, Q., and Bei, Y. (2012). A comparative study on proteomics of cerebral infarction with Liver-yang transforming into wind syndrome and Wind-stirring due to yin-deficiency syndrome. Journal of Hunan University of traditional Chinese Medicine*.* 32(7), 54-57 29. Wang, W., Huang, J., Tan, J., Chen, Q., Xie, J., Yang, J., Yan, Y., and Gu, L. (2016). Toll like receptor 5 polymorphism is significantly associated with ischemic stroke with Wind-phlegm stagnation syndrome. Liaoning Journal of traditional Chinese Medicine*.* 43(12), 2495-2497 30. Wei, L., Xie, D., Zhang, J., Bao, Y., Huang, X., Jin, S., Zhou, L., Yang, B., Zhang, R., Wei, T., and Chen, H. (2019). Investigation of the correlation between traditional Chinese medicine syndromes and MTHFR C677T polymorphism of methylenetetrahydrofolate reductase in post-stroke cognitive impairment. Journal of Liaoning University of Traditional Chinese Medicine. 21(2), 95-98 31. Xiao, M., Liang, Q., Xiong, X., Zeng, N., Qu, J., Zhang, Y., Chen, J., Liang, X., Zhao, Y., Yang, B., and Fan, R. (2008). Study of peripheral blood mononuclear cells of hypertension intracerebral hemorrhage patients with Liver-yang transforming into wind syndrome by proteomics technology. Practical Preventive Medicine*.* 15(3), 623-627 32. Xie, J., Gu, L., Chen, Q., Wu, G., Yan, Y., and Su, L. (2013). Correlation Study on 12p13 single nucleotide polymorphism rs12425791 and traditional Chinese medicine syndromes in ischemic stroke patients of the Han Chinese. Chinese Journal of Integrated Traditional and Western Medicine*.* 33(1), 47-50 33. Xiong, X., Chen, J., Liang, Q., Fan, R., Zeng, Q., Qu, J., Xiao, M., and Zhang, Y. (2011). Proteomics study on the essence of Liver-yang transforming into wind syndrome. Chinese Journal of Integrated Traditional and Western Medicine*.* 31(7), 913-920 34. Xiong, X., Liang, Q., Hou, J., Chen, J., Liu, A., Yan, D., and Guan, Y. (2007). Study on serum proteomics of hypertensive intracerebral hemorrhage patients With Liver-yang transforming into wind syndrome and Hyperactive liver-yang syndrome. Practical Preventive Medicine*.* 14(6), 1649-1652 35. Yang, B., Liang, Q., Xiong, X., Chen, J., and Xiao, M. (2014). A proteomic comparative study of hypertensive cerebral hemorrhage in acute stage of Liver-yang transforming into wind syndrome and in recovery stage of Wind-stirring due to yin-deficiency syndrome. Journal of Hunan University of Traditional Chinese Medicine*.* 34(10), 34-38+65 36. Yang, X., Gao, J., Zhang, N., Yang, S., Yang, D., Yu, M., Tian, T., and Li, G. (2019). Biomarkers of the pathogenesis of Stasis-heat syndrome in acute intracerebral hemorrhage based on the plasma differential metabonomics. Modernization of Traditional Chinese Medicine and Materia Materia-World Science and Technology*.* 21(10), 2062-2072 37. Zeng, N., Liang, Q., Xiong, X., Qu, J., Xiao, M., Zhang, Y., Liang, X., Zhao, Y., Yang, B., and Liu, W. (2008). Proteromics analysis and identification on peripheral blood lymphocyte of Liver-yang transforming into wind syndrome of cerebral infarction. Chinese Journal of Information on Traditional Chinese Medicine*.* 15(4), 11-15 38. Zhang, N., Tian, T., Yu, M., and Li, G. (2019). Differential proteomics analysis of pathogenic unit of Stasis-heat syndrome for acute intracerebral hemorrhage. Chinese Journal of Integrated Traditional and Western Medicine*.* 39(6), 675-680 39. Zhang, Y., Wang, Q., Ding, Q., Wang, S., Zhu, K., Wang, J., Wang, H., Ji, G., Chen, Y., and Wu, J. (2020). Correlation between CYP2C19 gene polymorphism and traditional Chinese medicine syndromes distribution in 70 patients with cerebral infarction. Chinese Journal of Hospital Pharmacy*.* 40(9), 1033-1037 40. Zhao, F., Huang, Y., Wu, M., Li, G., and Wu, M. (2018). Expressions of spectrum of inflammation-related and clotting-related genes in hemorrhagic stroke patients with Stasis-heat syndrome. J. Tradit. Chin. Med. 59(20), 1753-1757 41. Zhao, H., Liu, P., Xu, C., Li, G., Gao, L., and Luo, Y. (2019). Unique MicroRNAs Signature of Lymphocyte of Yang and Yin Syndromes in Acute Ischemic Stroke Patients. Chin. J. Integr. Med. 25(8), 590-597. doi: 10.1007/s11655-018-2843-3 42. Zhao, Y., Liang, Q., Xiong, X., Fan, R., Liang, X., Yang, B., and Guan, Y. (2008). Effect of Zhengan Xifeng decoction on proteomics of peripheral blood mononuclear cell in intracerebral hemorrhage patients with Liver-yang transforming into wind syndrome. China Journal of Traditional Chinese Medicine and Pharmacy*.* 23(10), 885-889 43. Zhu, Q., Wu, G., Li, G., Meng, S., Liao, W., and Cai, Y. (2019). Association of HDAC9 gene single nucleotide polymorphism locus rs2240419 with ischemic stroke and susceptibility to traditional Chinese medicine syndromes in Guangdong Han Chinese. Chinese Journal of Clinicians*.* 47(1), 35-40 |
| --- |
